# Measles virus and cytomegalovirus co-infection, in a child with recent SARS-CoV-2 infection, during COVID-19 pandemic: a case report

**DOI:** 10.1186/s12985-023-02099-8

**Published:** 2023-07-19

**Authors:** Giulia Piccirilli, Monia Gennari, Liliana Gabrielli, Marta Leone, Eva Caterina Borgatti, Alessia Cantiani, Federica Lanna, Fabio Magurano, Melissa Baggieri, Antonella Marangoni, Marcello Lanari, Tiziana Lazzarotto

**Affiliations:** 1 Microbiology Unit, IRCCS Azienda Ospedaliero-Universitaria di Bologna, Bologna, 40138 Italy; 2 Pediatric Emergency Unit, IRCCS Azienda Ospedaliero-Universitaria di Bologna, Bologna, 40138 Italy; 3 Department of Medical and Surgical Sciences (DIMEC), University of Bologna, Bologna, 40138 Italy; 4 Department of Infectious Diseases, National Measles Reference Laboratory-WHO/LabNet, Istituto Superiore di Sanità (ISS), Rome, 00165 Italy

**Keywords:** Measles virus, Cytomegalovirus, SARS-CoV-2, Case report

## Abstract

Measles virus (MV) and cytomegalovirus (CMV) may cause pediatric infection. We report the first described case of MV and CMV co-infection in an unvaccinated 13-mo-old girl, with a recent severe acute respiratory syndrome coronavirus 2 (SARS-CoV-2) infection, occurred during coronavirus disease 2019 (COVID-19) pandemic. The COVID-19 pandemic context, combined with patient’s complex clinical scenario, presenting symptoms as persistent fever, diarrhea, vomiting, maculopapular rash and edema, in addition to high level of inflammatory markers, led to a suspicion of multisystemic inflammatory syndrome in children (MIS-C). The final diagnosis and the successfully management of the case, discharged after resolution of symptoms, was achieved by a proper virological diagnosis and a close two-way cooperation between pediatricians and clinical microbiologists. The report mainly highlights that awareness about measles should be raised in unvaccinated patients with consistent symptoms, even in the COVID-19 era.

## Introduction

Acquired cytomegalovirus (CMV) infection is common in infancy and generally asymptomatic in healthy children [1]. Conversely, despite the immunization programs have significantly reduced the incidence of measles, the serious related complications keep on occurring frequently in children under the age of 5 years [2]. During the coronavirus disease 2019 (COVID-19) pandemic, there was a significant drop in measles cases worldwide [3]. Here was reported the first described case of measles virus (MV) and CMV co-infection in an unvaccinated 13-mo-old girl, with a recent severe acute respiratory syndrome coronavirus 2 (SARS-CoV-2) infection, during COVID-19 pandemic.

## Case report

A 13-mo-old girl of Pakistani origin living with her family in Italy, travelled back from Pakistan to Italy on March 28, 2021.

On May 2, 2021 she was admitted to the Pediatric Emergency Unit, IRCCS Azienda Ospedaliero-Universitaria di Bologna, because of 3 days persistent fever. SARS-CoV-2 infection testing performed on nasopharyngeal swabs gave negative results by both antigenic and molecular assays. After clinical examination, the patient was discharged with diagnosis of upper respiratory tract inflammation with left acute otitis media to be treated with amoxicillin-clavulanate. Two days later, she came back to the hospital for persistent high fever (39.5 °C) and onset of vomiting, diarrhea and inappetence. She had no relevant past medical history: born by vaginal delivery after full-term gestation, she had normal psychomotor and neurological development. Routine childhood immunization was administered except for the measles, mumps, and rubella (MMR) vaccine, that she had not received yet.

The clinical examination revealed moderate dehydration, rhinitis, dry cough, conjunctivitis, hyperemic pharynx, tonsil hypertrophy, oral leukoplakia and hyperemic left tympanic membrane. She was eupnoic but pulmonary auscultation showed sour vesicular murmur spread over the upper respiratory tract. Cardiac auscultation was normal.

Laboratory findings showed elevated alanine aminotransferase (ALT), aspartate aminotransferase (AST), lactate dehydrogenase (LDH) levels and a slight rise of c-reactive protein (CRP) (Table 1). Microbiological examinations performed on stool samples were negative as well as the molecular test for SARS-CoV-2-RNA detection on nasopharyngeal swab (Table 2).

**Table 1:** Laboratory findings

Laboratory findings (normal levels)	04 May	5 May	6 May	7 May	9 May	11 May	15 May
Alanine aminotransferase U/L (< 45)	129	86	78	63	46	40	41
Aspartate aminotransferase (AST) U/L (< 60)	125	83	101	82	80	74	63
Lactate dehydrogenase U/L (180–430)	968	601	691	/	783	/	465
C-reactive protein mg/dL (< 0.50)	1.03	0.63	0.62	1.22	0.48	0.21	0.38
D-dimer mg/ L (< 0.55)	/	/	/	1.69	1.16	0.97	0.69
Gamma-glutamyl transferase U/L (< 22)	/	/	/	67	/	/	/
Interleukin-6 pg/mL (< 6.4)	/	/	/	37.7	8.7	/	/
Creatine kinase U/L (< 145)	/	/	/	/	337	/	/
Procalcitonin ng/mL (< 0.5)	/	/	/	1.8	0.3	0.1	/

/: not evaluated

**Table 2:** Microbiological examinations

	Peripheral blood	Respiratory sample	Feces	Urine	Saliva
**04 May**	NA	SARS-CoV-2-RNA (-)	Adenovirus Ag (-) Rotavirus Ag (-) Salmonella-Shigella-Campylobacter colture (-)	NA	NA
**05 May**	SARS-CoV-2-N IgG and IgM (+) SARS-CoV-2-S IgG (+) Enterovirus-RNA (-)	SARS-CoV-2-RNA (low +) Rhinovirus/Enterovirus-RNA (-) Chlamydia pneumonia-DNA (-) Mycoplasma pneumonia-DNA (-) Bordetella pertussis-DNA (-) influenza viruses-RNA (-) respiratory syncytial virus-RNA (-) parainfluenza viruses-RNA (-) human metapneumovirus-RNA (-) adenovirus-DNA (-) middle east respiratory syndrome coronavirus-RNA (-) coronavirus [HKU1, NL63, 229E, OC43]-RNA (-) Enterovirus-RNA (-)	NA	NA	NA
**06 May**	NA	SARS-CoV-2-RNA (low +)	NA	NA	NA
**07 May**	CMV-IgG (low +) CMV-IgM (+) MV-IgG (-) MV-IgM (BL) EBV VCA-IgG (-) EBV VCA-IgM (-) Brucella/Salmonella typhi Ab (-) HBs Ag (-) HCV IgG (-) HIV Ag/Ab (-) Blood colture (-)	SARS-CoV-2-RNA (-)	NA	NA	NA
**09 May**	CMV-DNA (+) [6,095] EBV-DNA (-)	NA	NA	CMV-DNA (+) [1,357] RV-RNA (-) MV-RNA (+)	RV-RNA (-) MV-RNA (+)
**11 May**	CMV-DNA (+) [4,416] RV IgG (+) RV IgM (-) MV IgG (+) MV IgM (+)	NA	NA	RV-RNA (-) MV-RNA (+)	RV-RNA (-) MV-RNA (+)

(-): negative; (+): positive; BL: borderline; NA: not analyzed; Ig: immunoglobulin; Ag: antigen; Ab: antibody; VCA: viral capsid antigen; SARS-CoV-2: severe acute respiratory syndrome coronavirus 2; MV: measles virus; RV: rubella virus; CMV: cytomegalovirus; EBV: Epstain-Barr virus; HBs: hepatitis B surface antigen; HCV: hepatitis C virus; HIV: human immunodeficiency virus; the CMV viral load (copies/mL) was reported in the square brackets

The patient was admitted to the Pediatric Emergency Department-Intensive Brief Observation (OBI) area, where intravenous rehydration and antiemetic (serotonin receptor antagonist) therapy was administered. The ibuprofen was used for management of fever. On May 5, negative result was obtained on nasopharyngeal aspirate by a multiplex polymerase chain reaction system for the detection of common respiratory pathogens. A low positive result for SARS-CoV-2-RNA in a new nasopharyngeal swab was found. This finding combined to positive antibody response against SARS-CoV-2, showed by positive results for both anti-nucleocapsid (N, 10.3 BAU/mL [border line range 0–1]) and anti spike (S, 30.4 BAU/mL [border line range 0-0.80]) antibodies, suggested a recent natural infection. On May 6, the patient was admitted in the Pediatric Unit with abdominal pain after palpation to the right lower quadrant, edema localized on eyelids, left wrist and hand at the site of peripheral venous catheter insertion. A mild maculopapular rash onset on face, neck and torso was also observed. The abdominal ultrasound examination revealed an acute cholecystitis. A chest radiography showed interstitial peribronchial thickening. Inhaled budesonide and intravenously ceftazidime (500 mg every 12 h) were used to treat airway inflammation and cholecystitis, respectively. An additional nasopharyngeal swab confirmed the low positive result for SARS-CoV-2-RNA. On May 7, positive serological results were also obtained for IgM against CMV (137 U/mL [border line range 20–22]) with low levels of anti-CMV IgG (21 U/mL [border line range 12–20]). Furthermore, a borderline value was obtained for anti-measles virus (MV) IgM (1.02 index [border line range 0.9–1.1]), while anti-MV IgG resulted negative (7.94 UA/mL [negative range 0–13]). Due to the persistent fever and the worsening of rash (spread to the trunk and extremities) and peripheral edema, an infectious disease specialist was consulted. A multisystemic inflammatory syndrome in children (MIS-C) related to COVID-19 was suspected on the basis of the overall clinical conditions and the higher levels of inflammatory markers detected (CRP: 1.22 mg/dL; procalcitonin: 1.8 ng/dl, interleukin-6: 37.7 pg/mL, D-dimer: 1.69 mg/L). Therefore, intravenous immunoglobulin (IV-Ig) therapy (2 g/kg) was administered in combination with acetylsalicylic acid as antiplatelet agent (5 mg/kg/once daily); antimicrobial therapy was switched to cefotaxime (50 mg/kg every 6 h). A cardiac involvement, frequently observed in MIS-C cases, was excluded by electrocardiogram and ultrasound. On May 9, the overall clinical conditions worsened and the existing treatment was implemented with methylprednisolone (2 mg/Kg/day) and heparin administered subcutaneously (100 UI/kg). For edema, both furosemide (5 mg 3 times daily) and albumin (1 g/Kg) were used. Meanwhile, the virological examinations revealed CMV-DNA in both blood and urine samples (6,095 copies/ml and 1,357 copies/ml, respectively), proving an active CMV infection. Furthermore, MV-RNA was detected in both saliva and urine samples. A second serological analysis showed IgM and IgG anti-MV positivity (6.10 index and 300 UA/mL, respectively); the IgG anti-MV levels were related to the IV-Ig treatment. These results, together with the previous findings, allowed to diagnose MV and CMV co-infection in a patient with a recent SARS-CoV-2 infection. The initial MIS-C hypothesis was excluded and the therapy based on corticosteroids, aspirin and heparin was stopped. Symptomatic treatment was maintained. On May 11, the patient’s conditions improved with gradual resolution of fever, peripheral edema, rash and conjunctivitis. She was discharged on May 15 after 11 days of hospitalization with resolution of symptoms, decreased levels of transaminases and inflammatory markers. The molecular characterization of MV strain involved was performed by our laboratory (belonging to MoRoNet, the National Network of Reference Laboratories for measles and rubella), amplifying and sequencing the region N terminal 450 (N-450) [4]. The results showed a MV strains belonging to B3 genotype. The result was confirmed by the National Reference Laboratory for Measles and Rubella (ISS, Rome), that submitted in the WHO database MeaNS the obtained sequence. Phylogenetic analysis was performed including international sequences from MeaNS. Results showed that the MV B3 strain involved in the infection was identical to strains identified a few weeks earlier in Pakistan, where the patient travelled approximately 4 weeks before the onset of symptoms leading to hospitalization. There were not any suspected measles cases notified among other family or Pakistani community members. Although epidemiological link was not found, the case was classified as correlated to an imported case on the basis of virological surveillance data.

## Discussion

Acquired CMV infection in healthy children is common and can asymptomatically proceed or cause a self limited mononucleosis-like syndrome. Unusual CMV-related manifestations have been also reported, such as protein-losing gastropathy presenting with vomiting, abdominal pain, and generalized edema [1, 5]. Measles is a very contagious infection with significant mortality and morbidity in children [6]. Unvaccinated young children are at the highest risk of measles and its complications [7]. In Italy, MMR vaccination is mandatory, starting with a first dose at 13 through 15 months of age [8, 9]. In this report, the 13-mo-old patient was not yet MMR immunized. At OBI admission, she presented symptoms consistent with different clinical conditions [5]. However, in a pandemic period like the past one, the positive result for SARS-CoV-2-RNA and antibodies anti-SARS-CoV-2 led to interpret those symptoms as suspected MIS-C, on the basis of World Health Organization (WHO) criteria [10]. This condition is a severe syndrome described from April 2020 in clusters of paediatric cases presenting multisystem inflammatory syndrome related to SARS-CoV-2 infection [11]. Both early identification and treatment of MIS-C are crucial to achieve good outcomes [12]. Our patient was hospitalized and was promptly treated, since no alternative microbial causes of systemic inflammation were found. The anticoagulant drug was administered for increased level of D-dimer (three times the upper limit of normal). Due to the worsening of clinical conditions, methylprednisolone was also added, as recommended for children not responding to early administration of IV-Ig alone [12].

In the next days, virological results showed CMV-DNA on blood and urine samples, revealing a primary acute CMV infection. Since CMV infection can potentially present with multisystem involvement, it should be considered in differential diagnosis with MIS-C, taking into account that severe CMV infections are described prevalently in immunocompromised children [13, 14]. In the meantime, the presence of MV-RNA in both saliva and urine samples showed an acute measles infection, excluding definitively MIS-C suspicion. There is no specific antiviral therapy for measles and the treatment received by the patient was prevalently supportive, in order to control fever and dehydration [15]. After 11 days of hospitalization, the patient was discharged with complete resolution of symptoms.

To our knowledge, this is the first described case of MV and CMV co-infection in a child with a recent SARS-CoV-2 infection. In this clinical scenario, it is difficult to establish the specific role that each viral agent may have played, including SARS-CoV-2. However, this report emphasized several important points. The first is that the awareness about measles should be raised in unvaccinated patients presenting consistent symptoms, even in the COIVD-19 era. In Italy, an efficient surveillance system related to the national plan for measles and congenital rubella elimination is available [16]. In this context, a sub-national reference laboratories network has been developed to guarantee proper virological diagnosis and surveillance for measles and rubella (MoRoNet), according to WHO standards. Only these laboratories are allowed to confirm or exclude suspected cases. In addition, the borderline value of IgM anti-MV detected on the first serum sample underlines that the optimal timing for specimens collection is pivotal for a proper measles diagnosis. Indeed, when a serum specimen is collected too early (≤ 3 days from rash onset), a MV-infected patient may not show specific IgM response yet, or the level of anti-MV IgM could be below the detection threshold. In these cases, the molecular diagnosis performed on urine and oral fluid samples is fundamental to confirm a measles case [17]. Another relevant point is the key role of virological surveillance, in order to discern between imported and autochthonous cases, with WHO’s goal to eliminate measles virus circulation. We identified a viral strain circulating in Pakistan during the first months of 2021, when our patient travelled there. Despite no epidemiological link was found, we might speculate that the patient was infected by other members of the Pakistani community members. From January to April 2021, an alarming rise of measles cases, mostly involving unvaccinated children, was reported in Pakistan (8,054 confirmed cases) [18], during the stay of our patient. In this geographical area, as in other countries, the suspension of immunization programs due to COVID-19 pandemic period could have trigged other public-health issues, including measles. Finally, we underline the importance of a close two-way collaboration between pediatricians and clinical microbiologists: only a strict cooperation strategy allowed a successful management of the patient presenting such a complex clinical scenario that required multiple differential diagnoses.

## Data Availability

Not applicable.
